# Interaction between effector and memory cd8+ t cells enhances melanoma adoptive immunotherapy

**DOI:** 10.1186/2051-1426-3-S2-P8

**Published:** 2015-11-04

**Authors:** Amanda Contreras, Andrew Tatar, Siddhartha Sen, Justin Meyers, Prakrithi Srinand, David Mahvi, Clifford Cho

**Affiliations:** 1 University of Wisconsin-Madison, Madison, WI, USA

## Introduction

Our laboratory has previously demonstrated that adoptive cell transfer (ACT) of melanoma-specific memory T cells (T_M_) results in a more potent local and systemic T cell response than ACT with melanoma-specific effector T cells (T_E_). However, we have also seen that T_M_ are not more cytotoxic than T_E_*in vitro*. We hypothesized that a combination of T_E+M_ ACT would have an additive effect compared to T_E_ and T_M_ ACT alone.

## Methods

C57BL/6 mice were inoculated with subcutaneous injections of B16F10 melanoma cells transfected to express low levels of the lymphocytic choriomeningitis virus (LCMV) peptide GP33 (B16GP33). GP33-specific T_E_, T_M_, or T_E+M_ ACT was administered seven days after tumor inoculation. Cultured GP33-specific T_E_, T_M_, or T_E+M_ were stimulated with GP33 or co-cultured with B16GP33 cells.

## Results

Combinatorial T_E+M_ ACT resulted in more potent suppression of *in vivo* B16GP33 melanoma growth compared to T_M_ or T_E_ ACT alone. T_E+M_ ACT resulted in slightly higher populations of total CD8+ TILs compared with T_M_ ACT. T_E+M_ ACT did not result in higher numbers of exogenously-derived transferred T cells; rather, combination ACT resulted in a profound induction of endogenous TILs. In addition, combination ACT induced the most potent systemic T cell response to tumor antigen. *In vitro*, T_E_ and T_M_ were comparable in their ability to inhibit of melanoma growth, but T_E+M_ was synergistic. This synergy was reproduced by applying conditioned media derived from activated T_M_ to co-culture wells containing T_E_. The addition of neutralizing IL-2 antibody negated this effect. T_E_ were more effective at inhibiting melanoma cell growth at early time points, but the strength of this inhibition diminished over time. In contrast, T_M_ became more effective at inhibiting melanoma growth over time.

## Conclusions

Overall, these data suggest that a synergistic interaction between T_E_ and T_M_ may promote combinatorial ACT's superior anti-tumor efficacy. Combinatorial ACT's strong endogenous T cell infiltration and systemic response indicates that the combination's synergistic effect is enhancing the host's immune system. Our *in vitro* results suggest that cytokines released by stimulated T_M_ may augment the local cytotoxicity of T_E_ and that the temporal differences in killing may enhance the combination's ability to inhibit tumor growth. Further investigations will be performed to understand the cellular and molecular mechanisms responsible for this clinically promising observation.

